# Platform for Active Vaccine Formulation Using a Two-Mode Enhancement Mechanism of Epitope Presentation by Pickering Emulsion

**DOI:** 10.1021/acsabm.2c00410

**Published:** 2022-08-01

**Authors:** Guy Mechrez, Karthik Ananth Mani, Abhijit Saha, Oded Lachman, Neta Luria, Ori Molad, Liliya Kotliarevski, Einat Zelinger, Elisheva Smith, Noga Yaakov, Dalia Shabashov Stone, Meital Reches, Aviv Dombrovsky

**Affiliations:** † Department of Food Science, Institute of Postharvest and Food Science, Agricultural Research Organization, The Volcani Institute, Rishon LeZion 7505101, Israel; ‡ Institute of Chemistry and The Center for Nanoscience and Nanotechnology, The Hebrew University of Jerusalem, Jerusalem 9190401, Israel; § Department of Plant Pathology and Weed Research, Institute of Plant Protection, Agricultural Research Organization, The Volcani Institute, Rishon LeZion 7505101, Israel; ∥ The Interdepartmental Equipment Unit, The Robert H. Smith Faculty of Agriculture, Food and Environment, The Hebrew University of Jerusalem, Rehovot 7610001, Israel; ⊥ Institute of Biochemistry, Food Science and Nutrition, The Robert H. Smith Faculty of Agriculture, Food and Environment, The Hebrew University of Jerusalem, Rehovot 7610001, Israel; # Pharmaseed Ltd., Ness Ziona 74047, Israel; ○ Department of Chemistry, SRM Institute of Science and Technology, Kattankulathur, Chennai 603203, India

**Keywords:** virus-like-particles, subunit vaccines, epitope-based vaccination, Pickering emulsion, plant virus

## Abstract

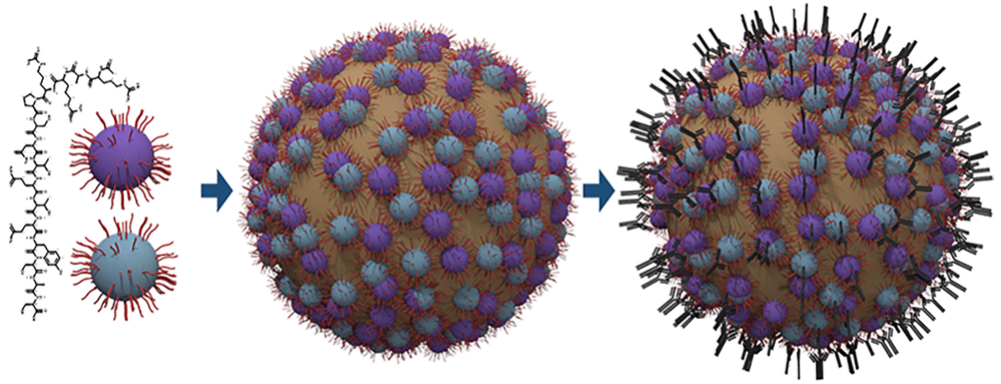

The efficiency of epitope-based vaccination (subunit vaccines) is tightly correlated with heterogeneity and the high density of epitope presentation, which maximizes the potential antigenic determinants. Here, we developed a two-mode platform for intensifying the epitope presentation of subunit vaccines. The two-mode epitope presentation enhancement includes a covalent attachment of high concentrations of SARS-CoV-2-S1 peptide epitope to the surface of virus-like-particles (VLPs) and the subsequent assembly of VLP/epitope conjugates on the oil droplet surface at an oil/water interface of an emulsion as Pickering stabilizers. The resultant emulsions were stable for weeks in ambient conditions, and our platform was challenged using the epitope of the SARS-CoV-2-S1 peptide that served as a model epitope in this study. *In vivo* assays showed that the αSARS-CoV-2-S1 immunoglobulin G (IgG) titers of the studied mouse antisera, developed against the SARS-CoV-2-S1 peptide under different epitope preparation conditions, showed an order of magnitude higher IgG titers in the studied VLP-based emulsions than epitopes dissolved in water and epitopes administered with an adjuvant, thereby confirming the efficacy of the formulation. This VLP-based Pickering emulsion platform is a fully synthetic approach that can be readily applied for vaccine development to a wide range of pathogens.

## Introduction

The recent emergence of the SARS-CoV-2 pandemic has emphasized the need for rapid vaccine development strategies, which can be readily adapted to pathogen variations and to specific pathogen-related needs. The current SARS-CoV-2 vaccine technologies can be classified into two main categories: (i) nucleic-acid-based vaccines, which include mRNAs and DNA plasmids that encode viral antigenic proteins produced by the host cells, as well as viral vector-based vaccines and attenuated live-viruses, and (ii) protein-based vaccines, which are based on the presentation of antigenic viral peptides, as well as inactivated whole virus and subunit vaccines.^1−6^

Subunit vaccines are based on the presentation of one or more viral antigens (e.g., proteins, peptides, and carbohydrate antigens) on a carrier that instigates an immune response without introducing the whole pathogen and without any host cell modifications; they potentially provide the safest vaccine technology that can be applied against SARS-CoV-2.^7−11^ One of the major challenges in developing subunit vaccine formulations is the ability to immobilize and expose high numbers of epitopes on the vaccine vector to stimulate a suitable immune response, which is required for efficient and successful vaccination. Therefore, the development of innovative approaches that could ensure a highly efficient immune response toward the antigenic subunits is challenging and important for improving the current clinical applications.^1,12−15^

**Figure 1:**
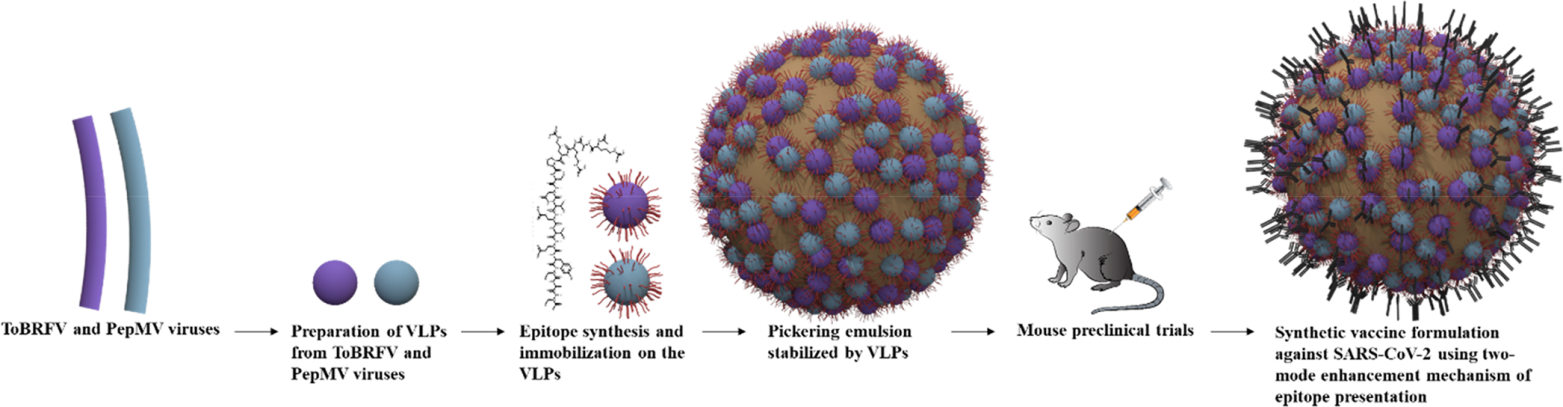
Schematic illustration of the two-mode enhancement mechanism for epitope presentation: (1) the immunogenic VLPs are prepared on the basis of ToBRFV and PepMV CPs; (2) the model epitope of SARS-CoV-2 is synthesized and covalently immobilized on the VLP surface, thereby exhibiting the first level of epitope concentration enhancement; (3) the VLP/epitope conjugates are then assembled at the oil/water interface of a Pickering emulsion to obtain a second level of epitope concentration enhancement; and (4) *in vivo* trials of the VLP/epitope-Pickering emulsions for immunogenicity in mice are conducted.

Here, we present a novel epitope display approach that engages viral-like particles (VLPs) assembled from the coat proteins (CPs) of two different plant virus genera, *Tobamovirus* and *Potexvirus*, as the immunogenic innocuous carrier for epitope display. Our formulation is fully synthetic and will open up the possibility to develop very safe and efficient vaccine systems. In this formulation, we have developed a two-mode epitope presentation enhancement mechanism that has significantly increased the immunogenicity of the studied model epitope compared with the control systems. The first level of enhancement of the epitope presentation intensity was achieved by covalent attachment of epitopes on the VLPs. The second enhancement level is obtained by the assembly of the VLP/epitope conjugates on the surface of oil droplets as Pickering stabilizers of oil/water emulsions (see a schematic in Figure 1). Our novel platform enhances the epitope density (surface concentration) on the vaccine vessel in comparison with the current state-of-the-art epitope presentation methods. These methods include standard emulsions,^16^ liposomes,^17^ cell-wall-localized epitopes,^18^ nanotechnology-based approaches,^19,20^ biodegradable polymers,^21,22^ antigen expression in transgenic plants,^23^ plasmid-based DNA vaccines,^24−26^ agonists,^27^ vector-based vaccines,^28^ and engineered dimers.^29^ Our approach can be easily modified to develop vaccines against many types of antigens by creating a large variety of antigenic peptides. The highly stable VLPs can be preserved at room temperature for months.

## Results and Discussion

A recent publication regarding SARS-CoV-2 revealed that the C-terminus of the spike glycoprotein (SG) contains an additional unique amino acid sequence that is absent in other coronaviruses.^30^ The authors suggested that this amino acid sequence is involved in the pathogenicity of the virus and, therefore, could be targeted for the development of antiviral immunity. This peptide sequence was used as a model epitope in this study to demonstrate the feasibility of our vaccine formulation to enhance the immunogenicity of a given epitope by two-level enhancement of the epitope presentation intensity.

The first epitope presentation enhancement level was obtained by covalent immobilization of peptide epitopes on the surface of immunogenic innocuous virus-like particles (VLPs) derived from the coat proteins (CPs) of two plant viruses, namely, tomato brown rugose fruit virus (ToBRFV) and Pepino mosaic virus (PepMV), which belong to two different genera: *Tobamovirus* and *Potexvirus*, respectively (Figure 1). It has recently been shown that ToBRFV and PepMV CPs induce rabbit immune responses when administered separately and result in high concentrations of specific antibodies toward each of these viruses.^31−33^ The second level of epitope concentration enhancement was obtained by assembling the VLP/epitope conjugates on the surface of paraffin oil droplets at the interface of paraffin-in-water emulsions as Pickering stabilizers (Figure 1).

Pickering emulsions are commonly formed by the self-assembly of colloidal particles at the interface between two immiscible liquids. The strong anchoring of the nanoparticles at the oil/water (o/w) interface is due to the partial wetting of the particles’ surface by both liquids.^34−39^ Importantly, it has been shown that Pickering emulsions are highly stable and could serve as adjuvants, thus enhancing the recruitment and activation of antigen-presenting cells.^40,41^ In our approach, we combined several principles of vaccine design that could provide a generic method for vaccine formulations to be potentially implemented in vaccines against a large variety of viruses or pathogens.

### Self-Assembly of VLPs

**Figure 2:**
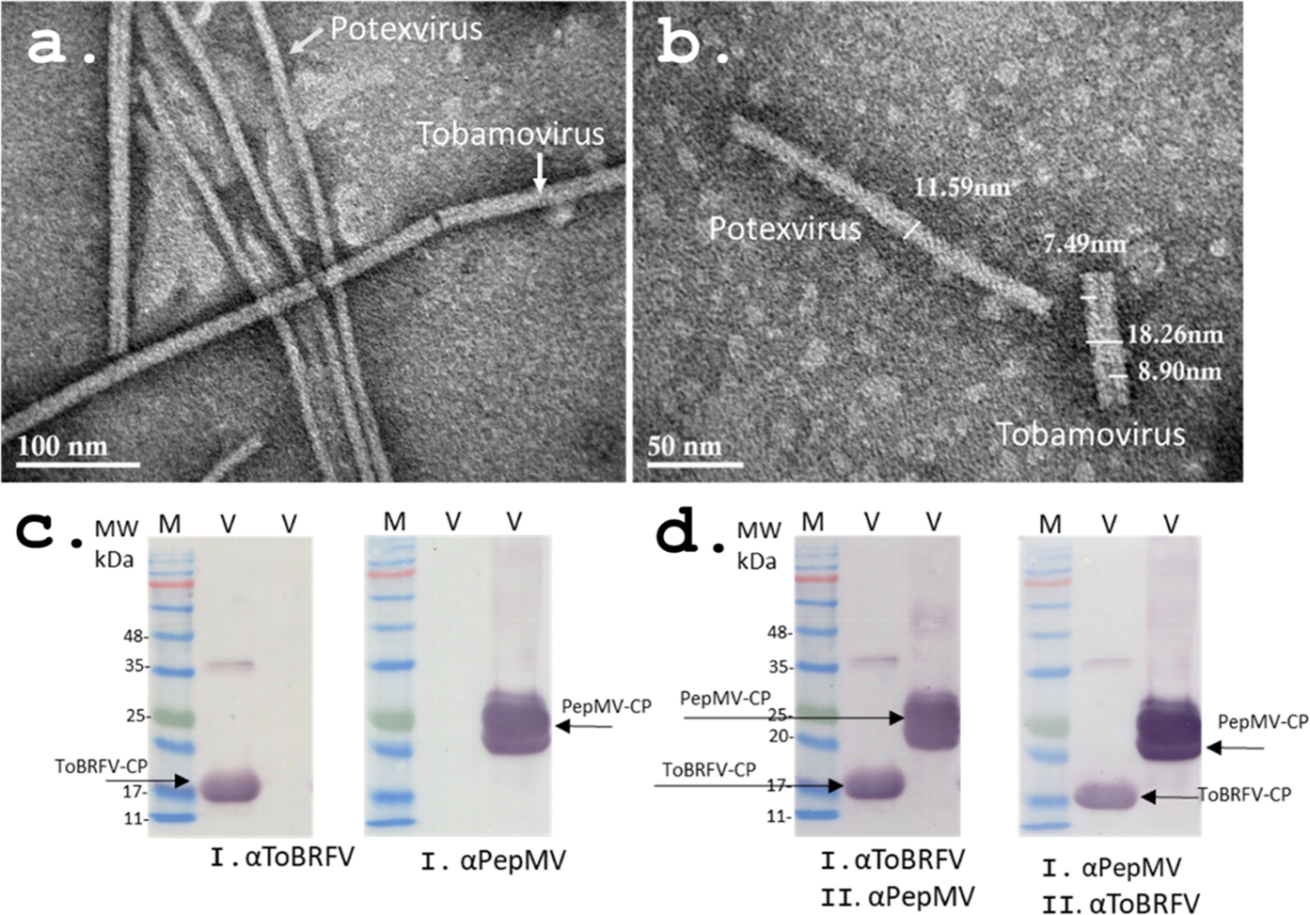
Purification assessment of ToBRFV and PepMV viral particles in viral preparation from coinfected tomato plants. (a,b) Transmission electron microscopy for visualizing virion particle morphologies. (c) Western blot analyses for detecting ToBRFV and PepMV coat proteins (CPs) in viral preparation from the coinfected tomato plants. (d) Sequential Western blot analyses for cross detection of ToBRFV and PepMV CPs on each of the analyzed viral preparations (M, molecular weight ladder; V, viral preparation).

We increased the heterogeneity of the epitope presentation and maximized the exposure of potential antigenic determinants by preparing a single type of VLP composed of two types of viral coat proteins. Before VLP assembly, two viruses belonging to two plant virus genera, *Tobamovirus* and *Potexvirus*, were purified from mixed infected tomato plants. The viral particles were purified using 100 g of symptomatic tomato fruits and leaves, as described by Luria et al.^31^Figure 2a,b presents the transmission electron microscopy (TEM) characterizations of the naturally occurring virus blend prepared from ToBRFV- and PepMV-infected symptomatic tomato plants. The rodlike and filamentous particle structures of ToBRFV and PepMV, respectively, can be visualized. The TEM data also shows that ToBRFV and PepMV are approximately 18 and 12 nm in their diameters, respectively. Western blot analyses showed the presence of ToBRFV and PepMV in the viral preparation from the coinfected tomato plants (Figure 2c,d). The ToBRFV CP of ∼17.5 kDa and PepMV CP of ∼26 kDa were specifically detected, and the presence of both viruses in each tested viral preparation was confirmed (Figure 2c,d).

**Figure 3:**
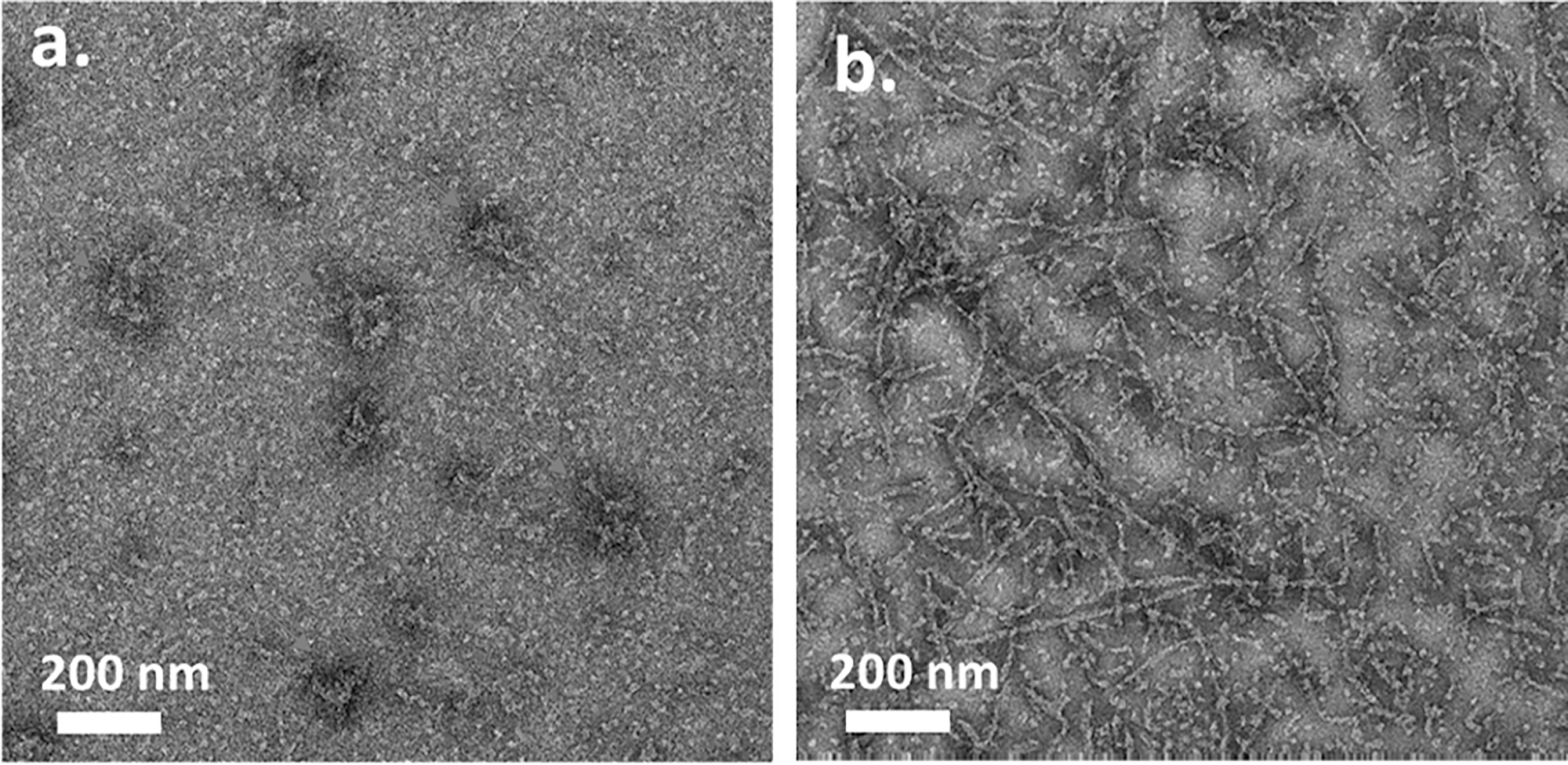
The stages of VLP preparation from ToBRFV and PepMV virions. (a) Heat denaturation of ToBRFV and PepMV after 2 min, 96 °C. Arrows indicate small aggregates of ToBRFV coat proteins. (b) RNase A is added, and the mixture is incubated at 96 °C for 5 min. Irregular and filamentous VLPs are visible.

The viral preparation samples, which contained a mixture of naturally occurring mixed infections of ToBRFV and PepMV in tomato plants, as seen in Figures 2c,d, were disassembled using a communally used process first described by Roger Hart in 1956.^42^ In this process, the viruses are incubated at 96 °C for 20 min for viral disassembly, and immediately afterward RNase A or H is added to the samples to degrade the two viral RNAs and to prevent the natural reassembly of the native viral particles, thereby allowing the new VLP structure to generate (Figure 3a,b). It is important to note that irregular and filamentous VLPs were assembled containing a mixture of two coat proteins from two different plant viruses (ToBRFV and PepMV).

### Synthesis of the VLP/Epitope Conjugates and Their Immobilization on the VLPs

**Figure 4:**
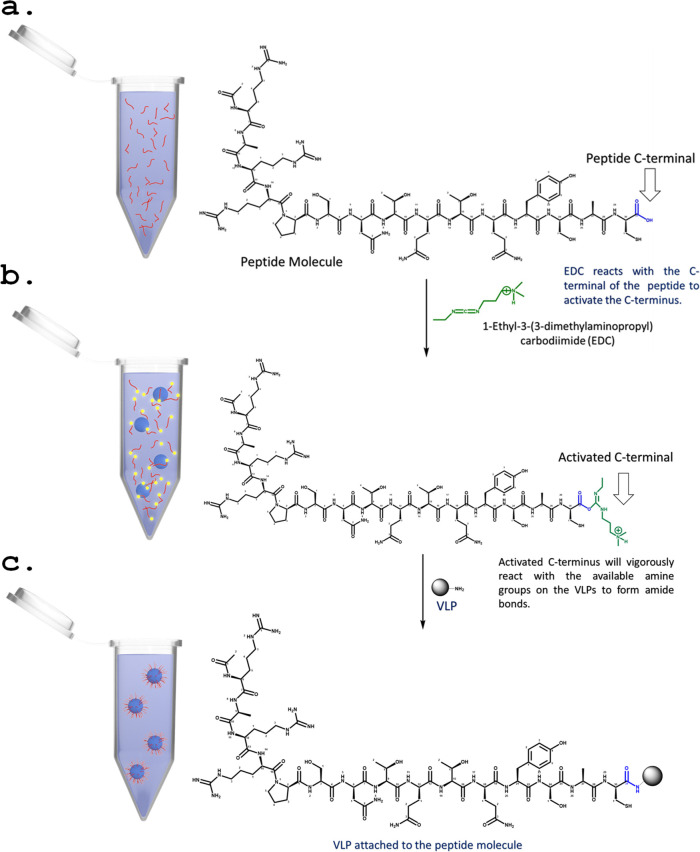
Schematic illustration of the conjugation reaction. (a) The studied peptide epitope is introduced to the EDC reagent, which reacts selectively with the C-terminus of the peptide. (b) Following the formation of the peptide/EDC derivative and its introduction to the VLPs, the activated C-terminus of the peptide/EDC derivative reacts with any available NH_2_ group on the surface of the VLPs to form an amide bond. (c) After the covalent immobilization of the peptide to the surface of the VLPs, the VLP/peptide conjugate is formed.

The amino acid sequence CASYQTQTNSPRRAR, which is unique to the spike glycoprotein of SARS-CoV-2,^30^ was used here as a model epitope for vaccine preparation against SARS-CoV-2. This sequence was recently confirmed as highly immunogenic.^32^ The epitope was synthesized by simple solid-state peptide synthesis (SSPS). The resulting synthetic peptide has an acetyl group at the N-terminus and a carboxyl at the C-termini that enables it to immobilize on the VLPs at the required directionality in accordance with the spike glycoprotein. Cross-linking chemistry by 1-ethyl-3-(3-(dimethylamino)propyl)-carbodiimide hydrochloride (EDC) was used to covalently immobilize the peptide on the VLPs by amidation through their carboxylic group, which reacts with the available amine groups on the VLPs. The coupling reagent EDC was used to covalently immobilize the peptide to the VLP by primarily reacting with the carboxyl groups to produce an amine-reactive O-acylisourea. This intermediate product reacted with the amino groups of the VLP to yield an amide bond, which formed the VLP/peptide–epitope conjugates and urea as a byproduct. This procedure has been widely utilized in many research fields and is referred to as one of the most cited chemical cross-linking procedures.^43−46^ A schematic illustration of the covalent immobilization of the peptide through its C-terminus to the amine groups of the VLPs is shown in Figure 4. The resulting VLP/epitope conjugates were purified by ultracentrifugation and served as stabilizers at the o/w interface of oil droplets in oil-in-water Pickering emulsions to further enhance the epitope presentation.

### Plant Virus VLPs Could Serve as Stabilizers of Pickering Emulsions

ToBRFV- and PepMV-derived VLPs were tested as effective stabilizers of oil-in-water Pickering emulsions using paraffin as the oil phase because of its well-established biocompatibility in many other vaccine formulations.^33,47^ Emulsions stabilized by VLPs were prepared by adding a known amount of paraffin oil (used as received) for the aqueous dispersion of VLPs (1.3 wt %) at o/w ratios of 20:80, 30:70, 40:60, and 50:50, respectively. We specifically chose to use paraffin oil because it is already being used in commercial vaccines. Prior to emulsification, the VLPs were dispersed in water via agitation in a vortex for 2 min. The emulsification was performed by ultrasonication in an ultrasonic probe for 10 min at an amplitude of 25%. The temperature measured around the sonication probe was lower than 80 °C to avoid any phase changes. Uniform emulsions were obtained at any of the aforementioned compositions.

**Figure 5:**
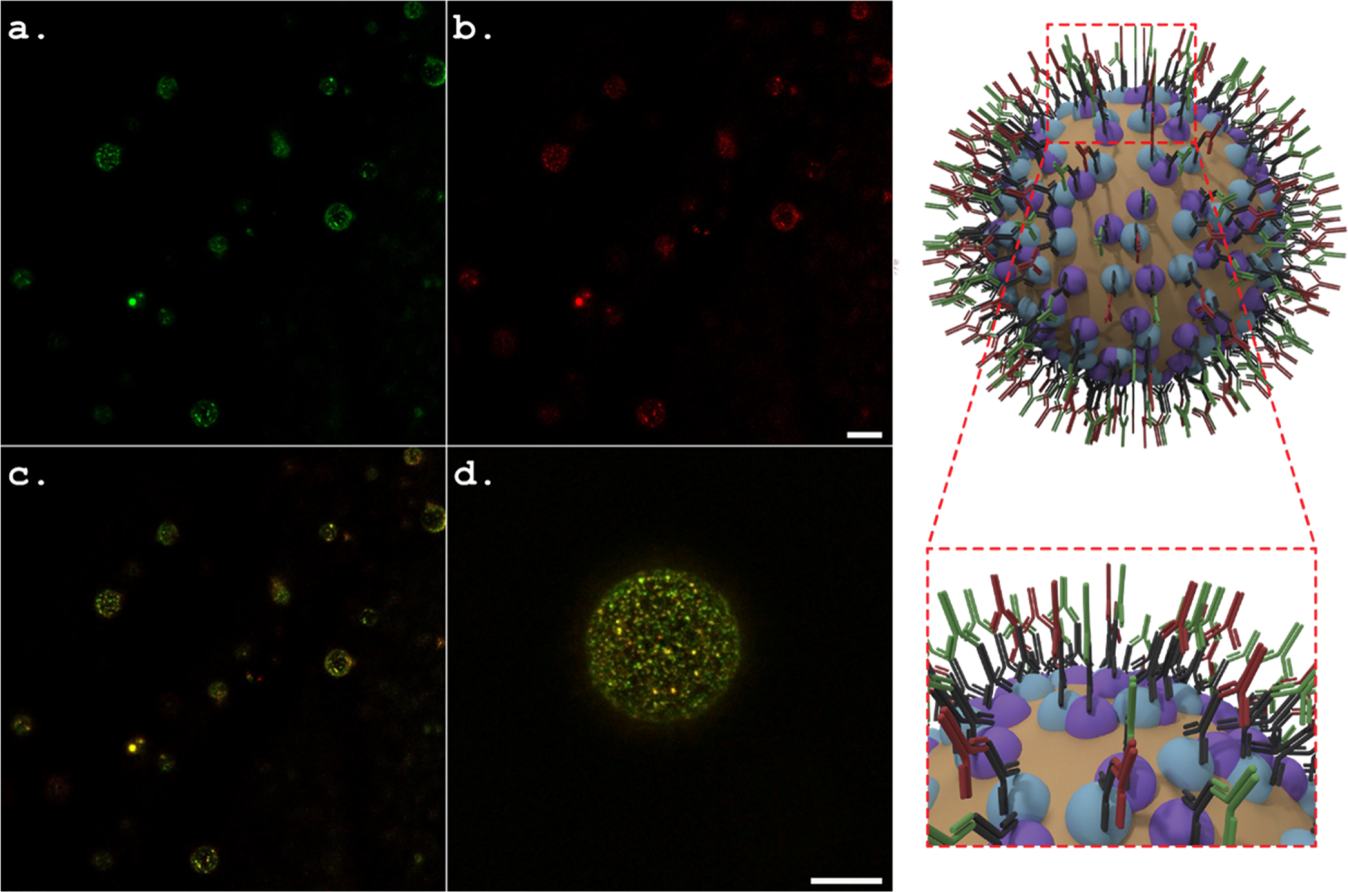
Characteristic confocal fluorescence microscopy images of 20:80 o/w Pickering emulsions stabilized by 1.3 wt % VLPs: (a) ToBRFV CP detection in Pickering emulsions subjected to specific fluorescent antibodies (Alexa Fluor 488) against ToBRFV using the green channel; (b) PepMV CP detection in Pickering emulsions subjected to specific fluorescent antibodies (Alexa Fluor 594) against PepMV using the red channel; and (c,d) low and high magnifications of the fluorescence signals in Pickering emulsions using combined green and red channels. A schematic illustration of the oil droplets in the VLP-stabilized Pickering emulsions is depicted on the right of the fluorescent images; the scale bar is identical for a and b and for c and d and represents 10 μm.

Visualization of the VLPs by immunofluorescence was carried out by subjecting the emulsions to ToBRFV and PepMV CP detection using specific primary antibodies, followed by secondary fluorescent antibodies Alexa Fluor 488 for ToBRFV (green, Figure 5a) and Alexa Fluor 594 for PepMV (red, Figure 5b). The fluorescent signals of both the ToBRFV and PepMV CPs were located at the interface of the oil droplets, which confirmed the assembly of both VLPs at the o/w interface and the stabilization of the Pickering emulsion. Visualization of the fluorescent signals using both the red and green channels showed that the two different VLP types homogeneously shared the interface (Figure 5c,d).

**Figure 6:**
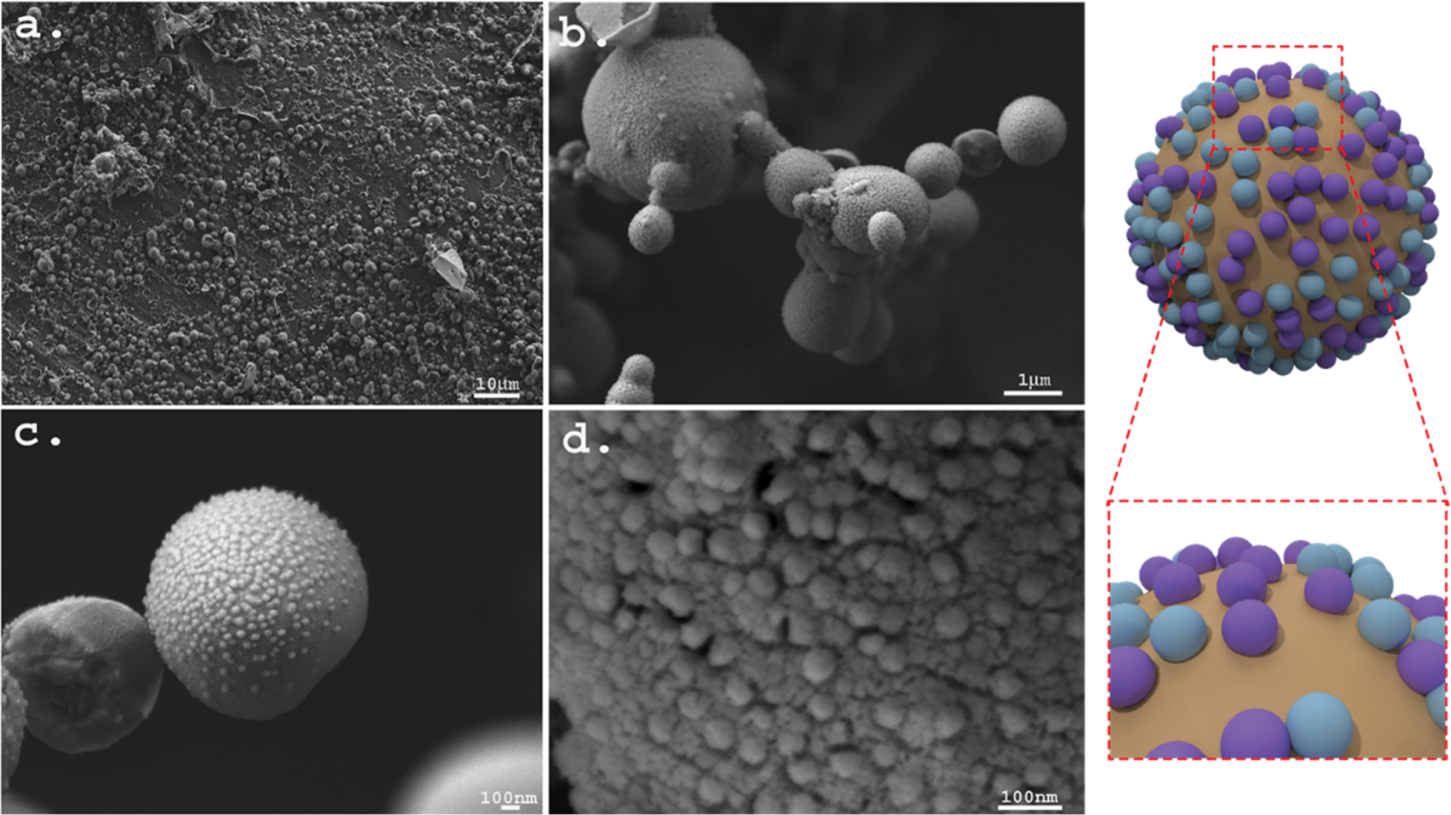
Characteristic cryogenic HRSEM micrographs of 20:80 o/w Pickering emulsions stabilized by 1.3 wt % VLPs. Pickering emulsions vitrified, fractured, and subsequently subjected to controlled sublimation for interface exposure were analyzed: (a,b) the characteristic basic structure of a Pickering emulsion and (c,d) higher magnification micrographs (10×) showing the presence of the VLPs at the o/w interface of the oil droplets. A schematic illustration of the oil droplets in the VLP-stabilized Pickering emulsions is depicted on the right of the micrographs.

The structure of the emulsions, and in particular, the nanostructure of the interface, was studied by cryo-HRSEM. The Pickering emulsions were vitrified using liquid nitrogen and then fractured. The vitrification procedure enabled us to directly observe the nanostructure of the interface since no structural changes took place during vitrification. In the second stage, the continuous phase of the emulsions (i.e., the water) was sublimated to reveal the interface, which made it possible to study its nanostructure.^38,39^Figure 6 depicts characteristic cryo-HRSEM micrographs of a 20:80 o/w emulsion stabilized by VLPs at a concentration of 1.3 wt %. A basic structure of a Pickering emulsion was observed, which confirmed the formation of a paraffin o/w emulsion (Figure 6a,b). At higher magnifications, a layer of nanoparticles decorating the surface of the oil droplets at the o/w interface was observed. The particle diameter range was 20–50 nm, which corresponds to the expected diameter of the VLPs (Figure 6c,d). The cryo-HRSEM direct observation results conclusively confirmed the successful assembly of the VLPs at the interface of the oil droplets. The stability of the emulsions was characterized by LUMiSizer, and the results are depicted in Figure S5. The values of the instability index are lower than 0.05, which shows the studied emulsions are highly stable.

### Development of Pickering Emulsions Stabilized by VLP/Epitope Conjugates

**Figure 7:**
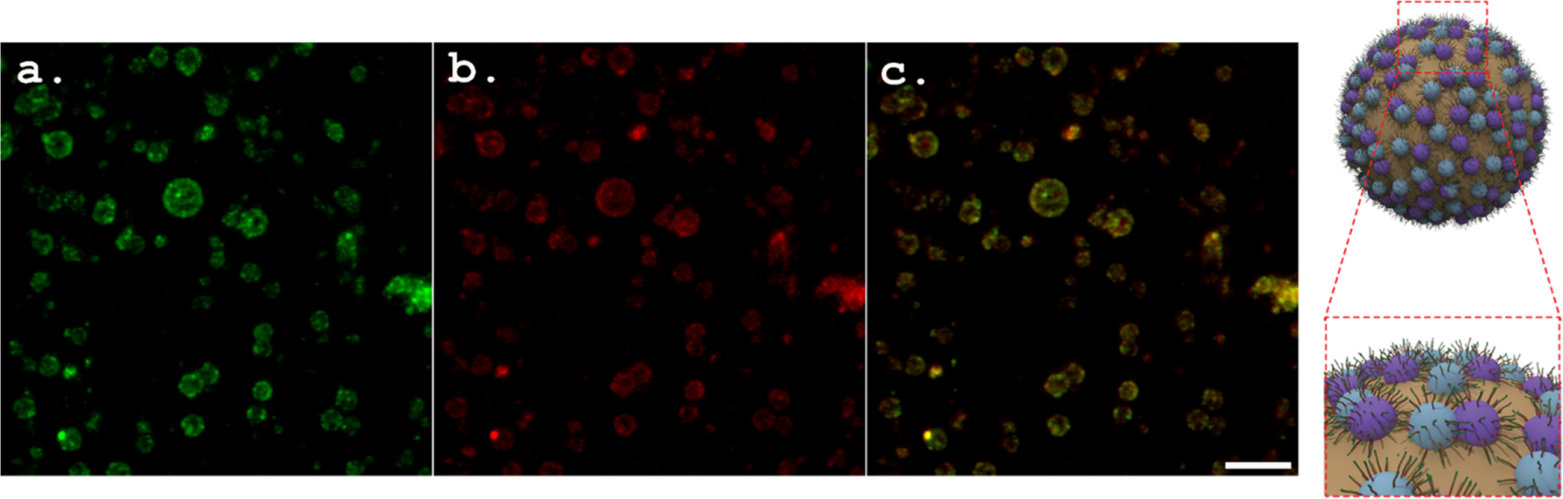
Characteristic confocal fluorescence microscopy images of 20:80 o/w Pickering emulsions stabilized by 1.3 wt % VLP/fluorescent epitope conjugates: (a) a [5(6)-FAM] labeled SARS-CoV-2 S1 epitope visualized on oil droplets with the green channel; (b) PepMV-CP detection using Alexa Fluor 594-specific fluorescent antibodies, visualized with the red channel; (c) colocalization of the green and red fluorescent signals (denoted in orange) visualized with both the green and red channels. A schematic illustration of the oil droplets in the Pickering emulsions stabilized by VLP/epitope conjugates is depicted on the right of the fluorescent images; the scale bar is for all images and represents 5 μm.

By successfully stabilizing paraffin oil-in-water Pickering emulsions, ToBRFV- and PepMV-derived VLPs could indicate that our newly designed platform would allow an enhanced presentation of SARS-CoV-2-S1 epitopes by using VLP/epitope conjugates as Pickering stabilizers. We confirmed our prediction by designing a unique fluorescent [5(6)-FAM]-labeled SARS-CoV-2-S1 peptide that was covalently immobilized on the VLPs. The VLP/[5(6)-FAM] peptide conjugates, dispersed in water at 1.3 wt %, were used as stabilizers of paraffin oil-in-water Pickering emulsions prepared by using four different o/w ratios: 20:80, 30:70, 40:60, and 50:50. The emulsification procedure and the compositions were identical to those used for the VLP-based emulsions. The resulting [5(6)-FAM]-labeled, conjugate-based Pickering emulsions were uniform at all of the studied o/w ratios. Visualization of fluorescent [5(6)-FAM]-labeled epitope/VLP conjugates in the Pickering emulsions with the green channel by confocal fluorescence microscopy clearly showed that the green fluorescence of the [5(6)-FAM]-labeled epitope was located at the o/w interface of the oil droplets (Figure 7a). The specific PepMV CP fluorescent signal, comprising the VLPs, was visualized with the red channel (Figure 7b); the colocalization of the red and green fluorescent signals was visualized with both the red and green channels (Figure 7c, denoted as orange signals). They confirmed that the peptide epitopes were covalently immobilized on the VLPs, which were assembled on the oil droplet surface. Importantly, this analysis, presented in Figure 7, overall confirmed the successful assembly of the VLP/epitope conjugates at the interface. The predicted enhanced pathogenic epitope was presented when our designed vaccine development formulation was used.

Positively charged particles stabilizing Pickering emulsions has been reported before in many studies. For example, Lactoferrin particles adsorb at the interface of oil-in-water emulsion droplets and form positively charged emulsion droplets that are stable over a wide range of pH values (from 3.0 to 7.0).^37^ The high specific surface area and hydrophobicity of the alum-packed oil-in-water Pickering emulsion were prepared for an enhanced Covid-19 vaccine adjuvant.^48^ In addition, it already has been confirmed in many studies that the positively charged particles can serve as robust Pickering stabilizers and lead to the assembly of a highly stable emulsion. Moreover, there is high flexibility in modifying the surface of the particles with a wide variety of peptides and other antigens. The surface properties of the Pickering emulsion stabilizer govern the emulsion stability and will determine the adsorption energy of the particles to the oil–water interface. Particles will assemble to the interface in accordance with their surface properties even if the particle–particle interaction is repulsion, such as in the case of positively charged nanoparticles. Indeed, as mentioned above, Pickering stabilizers can be positively or negatively charged and has been reported in the literature.^37,48^

### *In Vivo* Immunogenicity Assay of the Studied VLP/Epitope-Based Emulsions

We employed a standard Balb/C mouse vaccination scheme to evaluate the immunogenicity of the studied VLP/epitope-based emulsions. Briefly, blood samples were collected from the mice, and the sera were tested for the detection of immunoglobulin G (IgG) antibodies against the peptide antigen using ELISA. Three different dilutions of the serum were studied: 1:1000, 1:10 000, and 1:50 000.

**Figure 8:**
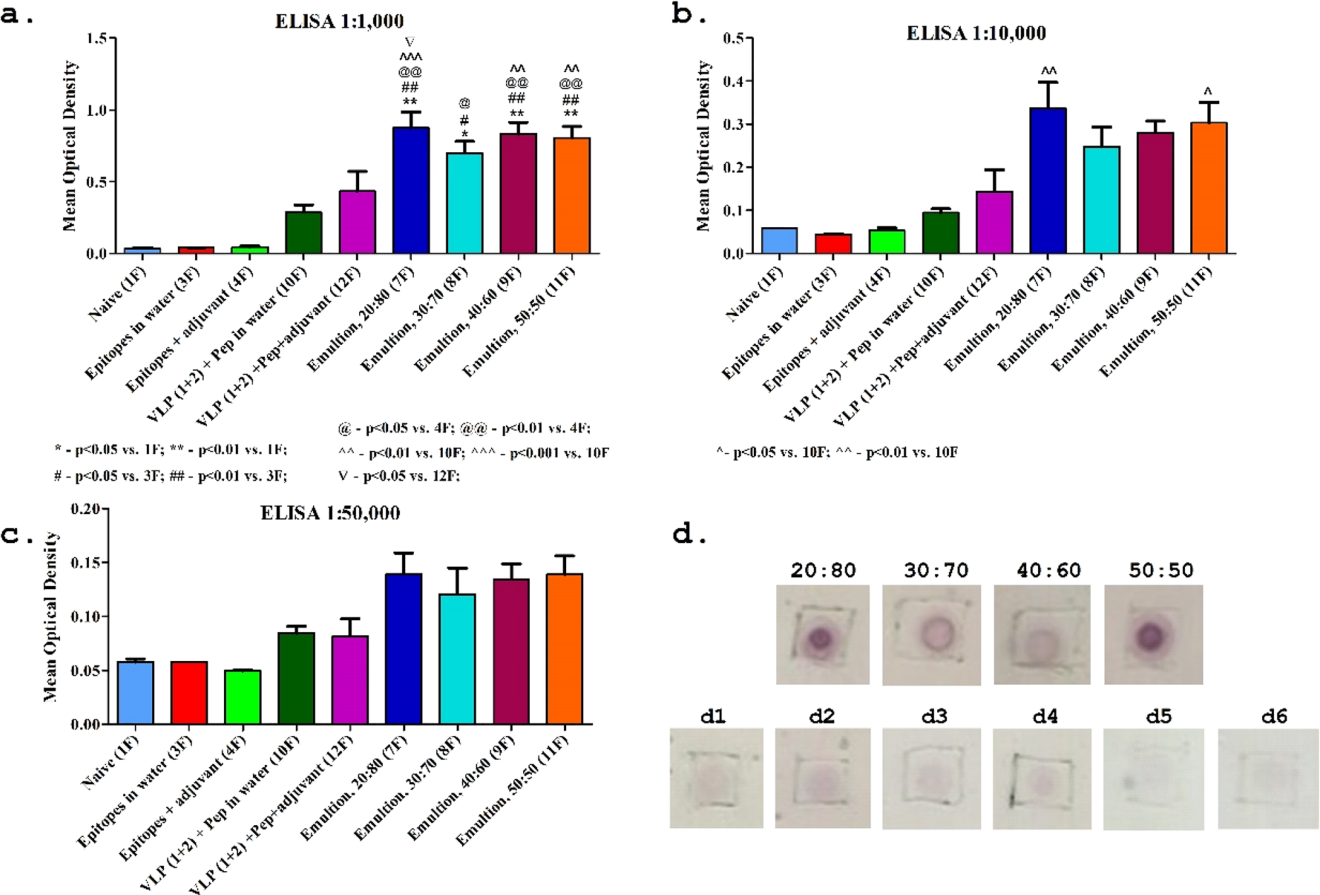
Immunization efficiencies and the specificity of antisera developed in mice vaccinated by VLP/epitope-based Pickering emulsions designed against SARS-CoV-2-S1 peptide. (a–c) ELISA tests of antisera produced in mice in response to various vaccine preparations against SARS-CoV-2-S1 peptide. (d) Dot blot analyses of mouse antisera developed against the synthetic SARS-CoV-2-S1 peptide presented by the VLP/epitope-based Pickering emulations and controls. The first row depicts the blotted membranes of mouse sera exposed to the VLP/epitope Pickering emulsions prepared by using o/w ratios of 20:80, 30:70, 40:60, and 50:50. The second row depicts (d1) naïve mouse sera, (d2) VLPs (consisting of ToBRFV and PepMV) dissolved in water, (d3) peptide epitopes dissolved in water, (d4) peptide epitopes administered with adjuvants, (d5) alkaline phosphatase reagent control, and (d6) a secondary antibody control.

The αSARS-CoV-2-S1 IgG titers of the studied mouse antisera, developed against the SARS-CoV-2-S1 peptide under different epitope preparation conditions, showed an order of magnitude higher IgG titers in the studied VLP-based emulsions than epitopes dissolved in water and epitopes administered with an adjuvant (Figure 8a) when comparing treatments 3F and 4F to 10F and 12F, respectively. Importantly, the assembly of VLP/epitope conjugates at the oil/water interface, which stabilized the Pickering emulsions, showed an additional 1.5–2-fold higher IgG titers than the nonassembled VLP/epitope conjugates (Figure 8a) when comparing the 10F treatment of aqueous dispersions of VLP/epitope conjugates with the Pickering emulsion treatments of 7F, 8F, 9F, and 11F. These results conclusively confirmed our ability to obtain a two-mode enhancement of the SARS-CoV-2-S1 epitope presentation for developing a new subunit vaccine formulation against SARS-CoV-2 (Figure 8a,b). The specificity of the mouse antisera produced in the vaccinated mice against the SARS-CoV-2-S1 peptide was confirmed using dot blot analyses of the various antisera obtained by VLP/epitope-stabilized Pickering emulsions prepared using four different o/w ratios: 20:80, 30:70, 40:60, and 50:50. The results clearly indicate a higher production rate of the αSARS-CoV-2-S1 peptide^15^ in the studied emulsions (Figure 8d) when compared with epitopes dissolved in water or epitopes administered with an adjuvant (Figure 8d3,d4, respectively). These results conclusively confirmed that our two-mode enhancement mechanism of SARS-CoV-2-S1 epitope presentation could serve as an efficient vaccine. However, applying this model for specific immunization against SARS-CoV-2 requires a combination of several epitopes in our described vaccine development platform.

Analysis of the potential neutralizing effect of the generated antibodies against SARS-CoV-2 was not performed in the current study, as the objective was to exhibit the feasibility of our generic vaccine formulation to enhance the immunogenicity of a given epitope on the basis of the two-level enhancement mechanism of epitope presentation intensity.

## Conclusions

In this study, we presented a novel technology for enhancing the presentation intensity of epitopes in subunit vaccines. The technology is fully synthetic and does not involve weak antigens, DNA, or RNA vectors. For this purpose, we employed a VLP immunogenic innocuous carrier for SARS-CoV-2-S1 epitope presentation at a high intensity, which showed a successful immunization against SARS-CoV-2-S1 in mice. The high intensity of the epitope presentation was achieved by a two-mode enhancement mechanism. The first mode used a high epitope concentration of SARS-CoV-2-S1 peptide epitope covalently immobilized on the surface of highly stable *Tobamovirus*/*Potexvirus*-derived VLPs. The second mode was achieved by assembling the VLP/epitope conjugates on the surface of oil droplets at an oil/water interface of an emulsion as Pickering stabilizers (schematic illustration of the platform is depicted in Figure 1). The two viral genera sources enhanced the heterogeneity of the presented epitopes. This two-mode epitope display system induced a higher IgG titer in mice than the classical adjuvant-associated immunization.

The highly stable ToBRFV and PepMV VLPs open up the possibility to develop safe vaccine technologies with improved efficiency and shelf life against SARS-CoV-2. The emulations we studied were stable for weeks in ambient conditions. The described platform is highly flexible, and by using multiple epitopes, it can be easily applied and extended for immunization against a wide range of pathogen epitopes.

## Methods

### Chemicals and Buffers

All Fmoc-protected amino acids, Wang resin, and hexafluorophosphate azabenzotriazole tetramethyl uronium (HATU) were purchased from Matrix Innovation (Quebec, Canada). N,N′-dimethylformamide (DMF), dichloromethane (DCM), N,N′-diisopropylethylamine (DIPEA), piperidine, methanol, trifluoroacetic acid (TFA), diethyl ether, and ethanol were purchased from Bio-Lab (Jerusalem, Israel). Triisopropylsilane (TIPS), thioanisole, 1,2-ethanedithiol (EDT), acetic anhydride, hydroxybenzotriazole (HOBT), N,N′-diisopropylcarbodiimide (DIC), 5(6)-carboxyfluorescein [5(6)-FAM], and phenol were purchased from Sigma-Aldrich (St. Louis, Missouri, USA). Paraffin oil (puriss meets the analytical specification of Ph. Eur., BP, a viscous liquid), dimethylaminopropyl-N′-ethylcarbodiimide hydrochloride (EDC), and MES hydrate were purchased from Sigma-Aldrich. HPLC-grade water was purchased from Alfa Aesar and was used as received without further purification. The Sonics Vibra-cell ultrasonic liquid processor, Model-VCX 750 (Newtown, CT, USA) was used for ultrasonication.

### Synthesis of the Epitopes According to the SARS-CoV-2 Sequence

The sequence CASYQTQTNSPRRAR, which is unique to the spike glycoprotein of SARS-CoV-2, was used as a model epitope for SARS-CoV-2. The epitope was synthesized by solid-state peptide synthesis (SSPS). See the Supporting Information for the peptide synthesis and the purification protocol.

### Preparation of ToBRFV and PepMV Native VLPs

ToBRFV and PepMV coinfected tomato plants (leaves, fruits) were used as the starting material for virus purification, as described before.^49,50^ The obtained viral preparation was first visualized by transmission electron microscopy (TEM) to confirm the presence of the characteristic morphology of the two viral particles (*Tobamovirus* and *Potexvirus*). The viral preparation (10 mL) was then mixed with equal volumes of 0.01 M phosphate buffer pH 9.5 (v/v) and the purified viruses were disassembled by incubation at 96 °C for 20 min. RNase A (20 μL, 10 units per μL) was added, and the sample was incubated at 96 °C for an additional 5 min, followed by mild rotations at room temperature for 10 min to allow the natural assembly of the VLPs.

### Functionalization and Characterization of the VLPs with Synthetic Epitopes

Stock solutions of 57.51 mg of EDC were prepared separately in 10 mL of 0.1 M MES (pH 4.5–5) buffer. The carboxyl groups present in the peptide molecules reacted with the amine groups of the VLP in the presence of EDC to form an amide bond. In this study, the VLP/peptide ratio was 1:1000. Therefore, there was a large number of free carboxyl groups available for conjugation with the VLPs, and we can expect that all of the peptides were successfully conjugated into the VLPs. Next, 112.5 mg of the peptide molecules were added to a 60 mL mixture of 10 mL of the EDC and 50 mL of the VLP (12–15 mg/mL) dispersion. The solution was then mixed by shaker for 2 h at ambient temperature. Subsequently, the mixture was centrifuged and rinsed with MES buffer to remove excess reactants. EDC was used as a cross-linker to covalently immobilize the peptide molecule to the VLP by primarily reacting with the carboxyl groups, which produced an amine-reactive O-acylisourea. This intermediate product reacted with the amino groups of the VLP to yield an amide bond and form the VLP/peptide–epitope conjugates and urea as byproducts.^43−46^ The VLP/peptide–epitope conjugates were then dispersed again in water (pH ∼ 8.5) for further analysis. The same protocol was utilized for the synthesis of the VLP/fluorescent peptide conjugates.

### Preparation and Characterization of o/w Pickering Emulsions Stabilized by VLP/Epitope Conjugates

Oil-in-water emulsions stabilized by VLPs were prepared by adding a known amount of paraffin oil (used as received) to create a VLP aqueous dispersion (1.3 wt %) at o/w ratios of 20:80, 30:70, 40:60, and 50:50, respectively. Prior to emulsification, the VLPs were dispersed in distilled water (pH ∼ 8.5) via agitation in a vortex for 2 min. The emulsification was performed by ultrasonication in an ultrasonic probe for 10 min (the agitation speed was 25% amplitude, the ultrasonication power was 750 W, and the frequency of the ultrasonication was 20 kHz). The emulsions, which were stabilized by VLP/peptide–epitope conjugates and by VLP/fluorescent peptide, were prepared by the same aforementioned procedure using the same compositions.

### Confocal Laser Scanning Microscopy

Confocal images were collected on a Leica SP8 confocal microscope (Leica Microsystems CMS GmbH, Wetzlar, Germany) equipped with an inverted microscope fitted with a 40× HC PL APO CS2 (1.10 NA) water immersion objective. Excitations of 6-AF and Nile Red were from the 488 nm and the 552 nm laser lines of an OPS laser, respectively. The 1024 × 1024 pixels images were collected using Leica Application Suite X software (Leica Microsystems CMS GmbH, Wetzlar, Germany).

### Cryogenic-Field Emission Scanning Electron Microscopy

Cryogenic-field emission scanning electron microscopy (cryo-FESEM) analysis was performed on a JSM-7800F Schottky Field Emission Scanning Electron Microscope (Jeol, Ltd., Tokyo, Japan). Liquid nitrogen was used in all heat exchange units of the cryogenic system (Quorum PP3010, Quorum Technologies, Ltd., Laughton, United Kingdom). A small droplet of the freshly mixed emulsions was placed on the sample holder between two rivets, quickly frozen in liquid nitrogen for a few seconds, and then transferred to the preparation chamber where it was fractured (at −140 °C). The revealed fractured surface was sublimed at −90 °C for 10 min to eliminate any presence of condensed ice and then was coated with platinum. The temperature of the sample was kept constant at −140 °C. Images were acquired with a secondary electron (SE), a low electron detector (LED), or a backscattered electron (BSE) detector at an accelerating voltage of 1 to 15 kV and at a maiximum working distance of 10.1 mm.

### Immunofluorescence Detection of VLPs by Double Labeling

The presence of each virus coat protein in the oil–water interface was confirmed by immunofluorescence using specific primary ToBRFV and PepMV antibodies, followed by fluorescent secondary antibodies.

VLP samples (10–20 μL) were pipetted on polylysine-coated silicon chips, which were placed in 96-well plates, and incubated for 1 h at room temperature (RT). The unbound solution was removed and fixation was carried out for 1 h at RT using fixation buffer containing 4% (v/v) formaldehyde and 0.2% (v/v) glutaraldehyde in phosphate-buffered saline (PBS) pH 7.0. The fixation buffer was removed, and the samples were washed with PBS three times, 10 min each time, while rotating at 100 rpm at RT. Blocking was performed with 100 μL of PBS containing 2% (w/v) skim milk powder for 30 min at RT. The blocker was removed, and the samples were incubated with 100 μL of specific antisera against ToBRFV (1:4000 dilution in the PBS–milk solution) overnight at 4 °C while being shaken. The samples were washed three or four times with PBS pH 7.0 at RT for 10 min each, and 100 μL of the secondary antibody, goat antirabbit IgG [conjugated to Alexa Fluor 594 (Invitrogen, Carlsbad, CA, USA)] were added at a 1:1000 dilution in PBS and incubated for 3 h at 37 °C with agitation at 100 rpm. The samples were then washed three or four times with PBS pH 7.0 for 10 min each. All unbound ToBRFV antibodies were blocked with the addition of 100 μL of a high concentration of unlabeled AP-conjugated goat antirabbit antibodies (SIGMA, A9919, 1:100 dilution in PBS containing 2% nonfat milk), and the samples were incubated for 3 h at 37 °C. Washes (3–4×) with PBS pH 7.0 were carried out at RT for 10 min each with agitation. A blocking solution (100 μL of PBS containing 2% nonfat milk) was added, and the samples were incubated for 30 min at RT with agitation. The blocker was removed, and 100 μL of specific antisera against PepMV (1:8000 dilution in the PBS–milk solution) was added overnight at 4 °C while being shaken. Washes (3–4×) with PBS pH 7.0 were carried out at RT for 10 min each with agitation, and 100 μL of goat antirabbit IgG [conjugated to Alexa Fluor 488 (Invitrogen, Carlsbad, CA, USA)] was added at a 1:1000 dilution in PBS and incubated for 3 h at 37 °C with agitation at 100 rpm. Washes (3–4×) with PBS pH 7.0 were carried out at RT for 10 min each with agitation, and the samples were kept in 100 μL of PBS pH 7.0 in sealed plates at 4 °C.

### *In Vivo* Preclinical Trial in Mice

We evaluated the immunogenicity of the tested items by employing a standard vaccination scheme in Balb/C mice. Groups of seven female mice aged 6–7 weeks old were immunized via subcutaneous (SC) route with test items or controls at day 1 and boosted on days 14 and 28, with blood drawn before immunization and at termination. The samples were processed, and the sera were collected and analyzed for antiepitope reactions in a standard direct ELISA assay. This study was performed in compliance with “The Israel Animal Welfare Act” and followed “The Israel Board for Animal Experiments.” The following eight groups were immunized: (Epitope 1 only), (Epitope 1 in emulsion), (Epitope 2 only), (Epitope 2 in emulsion), (Epitope 3 only), (Epitope 3 in emulsion), (Emulsion only), and (Carrier VLP only).

### Evaluation Parameters

Morbidity and mortality were measured twice daily (once daily over the weekend). Body weight monitoring was measured during acclimation and then weekly thereafter. Blood draws were conducted at baseline and at termination (all mice). Blood processing was conducted by collecting blood from all mice at termination. Blood samples were processed into serum for detecting antibodies to the antigen by ELISA. Method development and antibody titer evaluation were conducted through the detection of the generation of antibodies (IgG) against the antigen by direct ELISA in the sera of immunized mice.

### IgG Quantification by Direct ELISA

The generation of antibodies was detected by ELISA. The purpose of this ELISA was to ascertain that the mice elicited an immune response against the antigen. On day 1, three 96-well ELISA plates were coated with 25 μL of VLP/peptide at 2.5 mg/mL (250 μg/100 μL) in carbonate/bicarbonate buffer (Sigma, Cat# C3041). The plates were incubated for 2.5 h at 37 °C. The coating solution was removed, and the plates were washed three times with wash solution (PBS/0.05% tween), with a 1 min incubation between washes. Next, 50 μL of blocking buffer (1% BSA in PBS) was added, and the plates were incubated overnight at 4 °C. On day 2, the blocking buffer was removed, and the plates were washed three times with wash solution (PBS/0.05% tween), with a 1 min incubation between washes. Finally, 25 μL of 1:1000, 1:10 000, and 1:50 000 serum samples (diluted in PBS/0.1% BSA) and blank (PBS/0.1% BSA only) were added, in duplicate, and the plates were incubated overnight at 4 °C. On day 3, the samples were removed and the plates were washed three times with wash solution (PBS/0.05% tween), with a 1 min incubation between washes. Next, 25 μL of secondary antibody (Peroxidase Affinipure Donkey Anti-Mouse IgG (H+L), Cat 715–035–151) was added, and the plates were incubated for 2 h at 37 °C. The samples were removed, and the plates were washed three times with wash solution (PBS/0.05% tween), with a 1 min incubation between washes. Next, 25 μL of TMB substrate was added to each well, and the plates were incubated for 15 min at room temperature or until the desired color was achieved. Finally, 25 μL of Stop Solution was added to each well before the plates were read at 450 nm using a microplate reader.

### Instability Analysis

The instability index of creaming separation was analyzed using LUMiSizer software (LUM GmbH, Berlin, Germany) and calculated with the included software (SepView 6.0; LUM). The polycarbonate cuvettes with a 2 mm optical path length were filled with 400 μL of 20:80, 30:70, 40:60, and 50:50 vol % of emulsions and were centrifuged in triplicate at 25 °C, simultaneously, at a centrifugal force of 600 rpm (33 g). The transmission profiles were captured at 865 nm throughout the cell for 6 h (200 profiles every 5 s, 100 profiles every 10s, 100 profiles every 30 s, and 600 profiles every 60 s).

### Statistical Analysis

The numerical results are given as the mean and standard error of the mean. Descriptive statistics and group comparisons of data were performed using a statistical analysis program (GraphPad Prism version 5.02 for Windows, GraphPad Software, San Diego, California, USA). One-way ANOVA followed by Bonferroni post-Hoc analysis was performed. A probability of 5% or less (*p* ≤ 0.05) was regarded as statistically significant.

## Supporting Information Available

Peptide synthesis, purification and characterization, ELISA protocol for serum samples, and instability index of Pickering emulsions (PDF)
