# Supplementary material for: Platform for Active Vaccine Formulation Using a Two-Mode Enhancement Mechanism of Epitope Presentation by Pickering Emulsion
Source: ACS Appl Bio Mater. 2022 Aug 1;5(8):3859–69. doi: 10.1021/acsabm.2c00410 (PMC9382630; doi:10.1021/acsabm.2c00410)
Supplement: Supplementary file 1 — mt2c00410_si_001.pdf [file mt2c00410_si_001.pdf]

Supporting Information

A platform for active vaccine formulation using a two-mode enhancement mechanism of epitope presentation by Pickering emulsion

Guy Mechrez*[1], Karthik Ananth Mani[1,5], Abhijit Saha[2,7], Oded Lachman[3], Neta Luria[3], Ori Molad[2],[3], Liliya Kotliarevski[1,5], Einat Zelinger[4], Elisheva Smith[3], Noga Yaakov[1], Dalia Shabashov Stone[6], Meital Reches[*2], and Aviv Dombrovsky[*3]

[1] Department of Food Science, Institute of Postharvest and Food Science, Agricultural Research Organization, The Volcani Institute, Rishon LeZion, 7505101, Israel

[2] Institute of Chemistry and The Center for Nanoscience and Nanotechnology, The Hebrew University of Jerusalem, Jerusalem, 9190401, Israel

[3] Department of Plant Pathology and Weed Research, Institute of Plant Protection, Agricultural Research Organization, The Volcani Institute, Rishon LeZion 7505101, Israel

[4] The Interdepartmental Equipment Unit, The Robert H. Smith Faculty of Agriculture, Food and Environment, The Hebrew University of Jerusalem, Rehovot 7610001, Israel.

[5] Institute of Biochemistry, Food Science and Nutrition, The Robert H. Smith Faculty of Agriculture, Food and Environment, The Hebrew University of Jerusalem, Rehovot 7610001, Israel.

[6] Pharmaseed Ltd., Ness Ziona, 74047, Israel.

[7] Department of Chemistry, SRM Institute of Science and Technology, Kattankulathur, Chennai 603203, India

*Corresponding authors:
E-mail address: guyme@volcani.agri.gov.il (G.M.)
E-mail address: meital.reches@mail.huji.ac.il (M.R.)
E-mail address: aviv@volcani.agri.gov.il (A.D.)

Peptide synthesis: The peptide, Ac-NH-Arg-Ala-Arg-Arg-Pro-Ser-Asn-Thr-Gln-Thr-Gln-Tyr-Ser-Ala-Cys-OH, and its 5(6)-FAM-labeled (Ex: 492 nm, Em: 514 nm) peptide were synthesized using wang resin having a substitution level of 0.83 mmol/g. Briefly, 300 mg of wang resin were swelled in a mixture of DMF and DCM (1:1) overnight prior to the synthesis. Each coupling reaction was performed using 5 equivalents of HATU as an activator, 5 equivalents of amino acids, and 10 equivalents

of DIPEA as the activator base. The concentration of amino acids and HATU in the coupling mixture was 0.2M. The arginine amino acid, which is after proline, was coupled two times. DMF was used as a solvent. The Fmoc deprotection was performed by using a 20% piperidine solution in DMF.

Acetyl protection at the N-terminal: The resin with free N-terminal of the peptide was treated with a mixture of acetic anhydride, HOBT, and DIPEA in DMF and stirred for 3 hours. It was performed twice to ensure complete N-terminal acetyl protection.

5(6)-FAM protection at the N-terminal: The resin with free N-terminal of the peptide was treated with a mixture of 5(6)-FAM, HOBT, and DIC in DMF and stirred for 48 hours. It was performed twice to ensure complete N-terminal 5(6)-FAM protection.

After the whole peptide was synthesized, the resin was washed with DMF (5 times), DCM (5 times), methanol (5 times), diethyl ether (5 times), and it was kept under a high vacuum pump for 4h to ensure complete drying. The resin containing peptide was treated with a cleavage cocktail containing TFA (92%), TIPS (1.5%), water (2%), thioanisole (1.5%), 1,2-ethanedithiol (1.5%), and phenol (1.5%) for 24 hours at room temperature under shaking. The cleavage solution (without resin) was collected into a 50 mL falcon tube and was evaporated to a minimum volume using a flow of N_2. The residue solution was poured into ice-cold diethyl ether for precipitation. It was then stored overnight at -20 °C. Next, it was centrifuged at 5000 rpm at 4 °C and the precipitate was dissolved in triple distilled water (TDW). Finally, the peptide was lyophilized to obtain a white solid powder.

Peptide purification and characterization: The peptide was purified by reverse phase preparative high-performance liquid chromatography (HPLC) using the Thermo Scientific Ultimate 3000 system with a C18 LC column (10 µm, 110 Å, 250 × 21.2

mm). A linear gradient (5% to 95%) flow of acetonitrile (with 0.1% TFA) with time in water (with 0.1% TFA) at a flow rate of 10 ml/min was used to elute the peptide; each fraction was characterized by electron spray ionization mass spectroscopy using an LCQ Fleet Ion Trap mass spectrometer (Thermo Fisher Scientific, Waltham, MA, USA). The purity was checked by analytical reversed phase high-performance liquid chromatography (HPLC) using a Waters e2695 separation module with a C18 LC column (5 μm, 110 Å, 250 × 4.6 mm). A linear gradient (5% to 95%) flow of acetonitrile (with 0.1% TFA) with time in water (with 0.1% TFA) at a flow rate of 1 ml/min was used to elute the peptide. UV detection at 220 nm was used to monitor the peptide flow through the column.

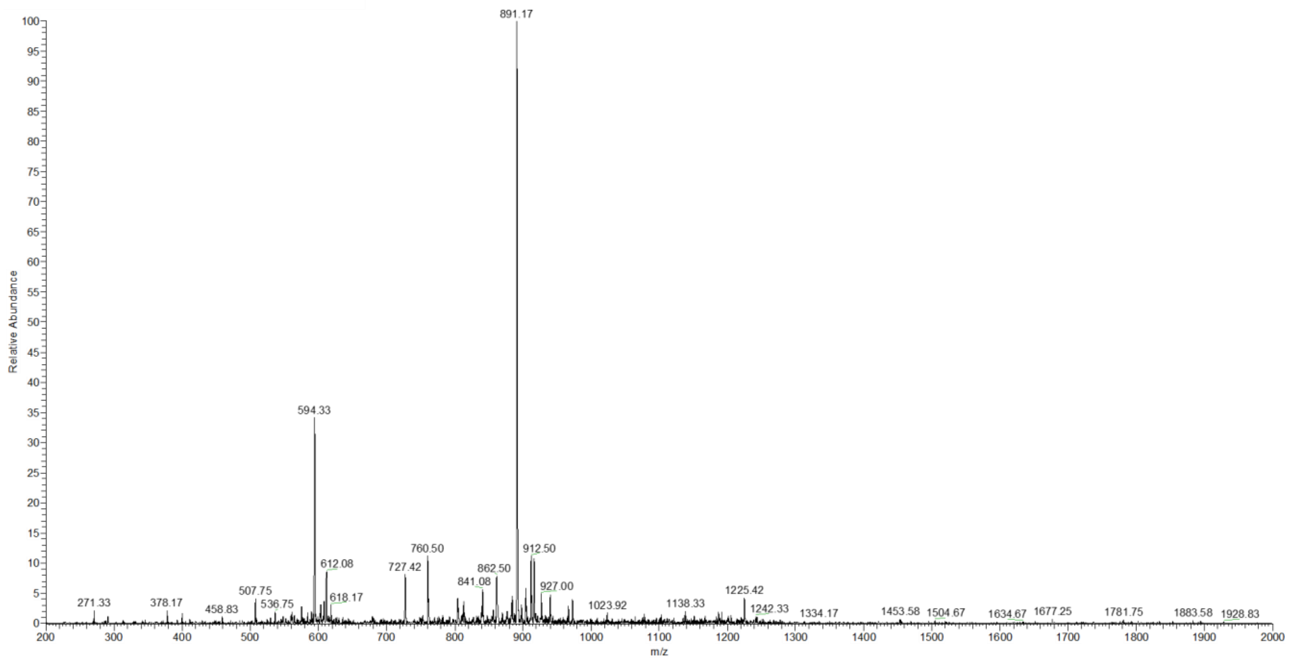

Fig. S1 The mass spectrum of the peptide (Ac-NH-Arg-Ala-Arg-Arg-Pro-Ser-Asn-Thr-Gln-Thr-Gln-Tyr-Ser-Ala-Cys-OH, MW: 1780). The peak at m/z = 891 and m/z = 594 are the m/2 and m/3 peaks of the peptide, respectively.

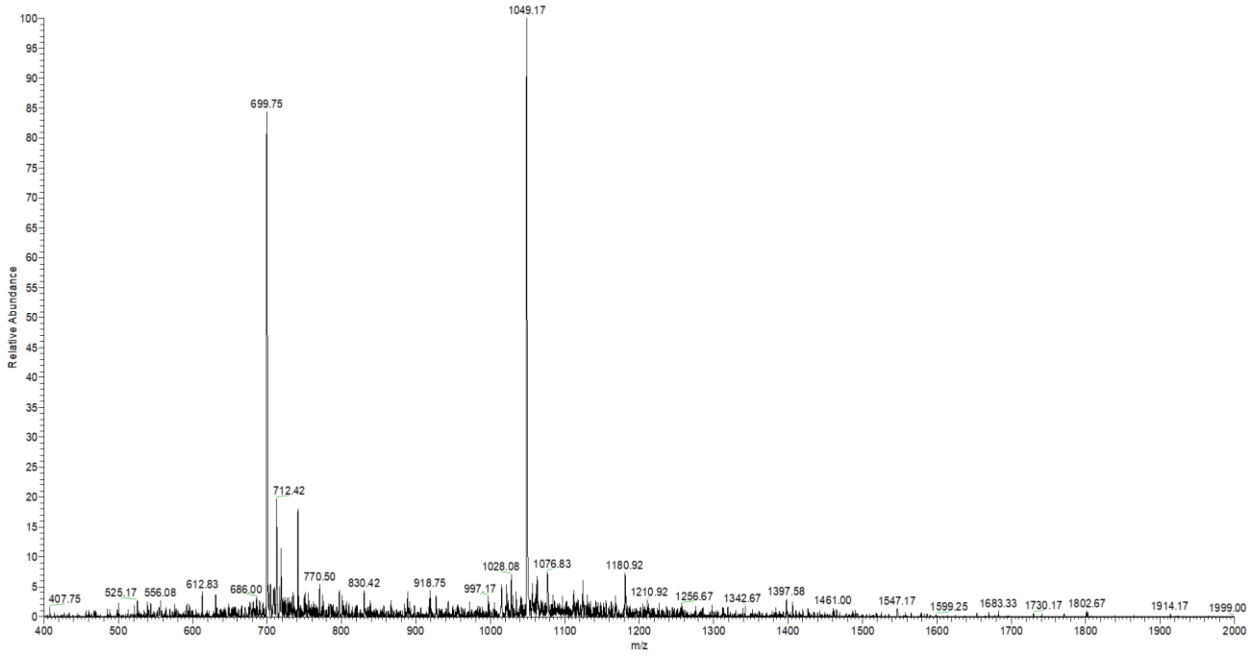

Fig. S2 The mass spectrum of the peptide [5(6)-FAM-NH-Arg-Ala-Arg-Arg-Pro-Ser-Asn-Thr-Gln-Thr-Gln-Tyr-Ser-Ala-Cys-OH, MW: 2097]. The peak at m/z = 1049 and m/z = 699 are the m/2 and m/3 peaks of the peptide, respectively.

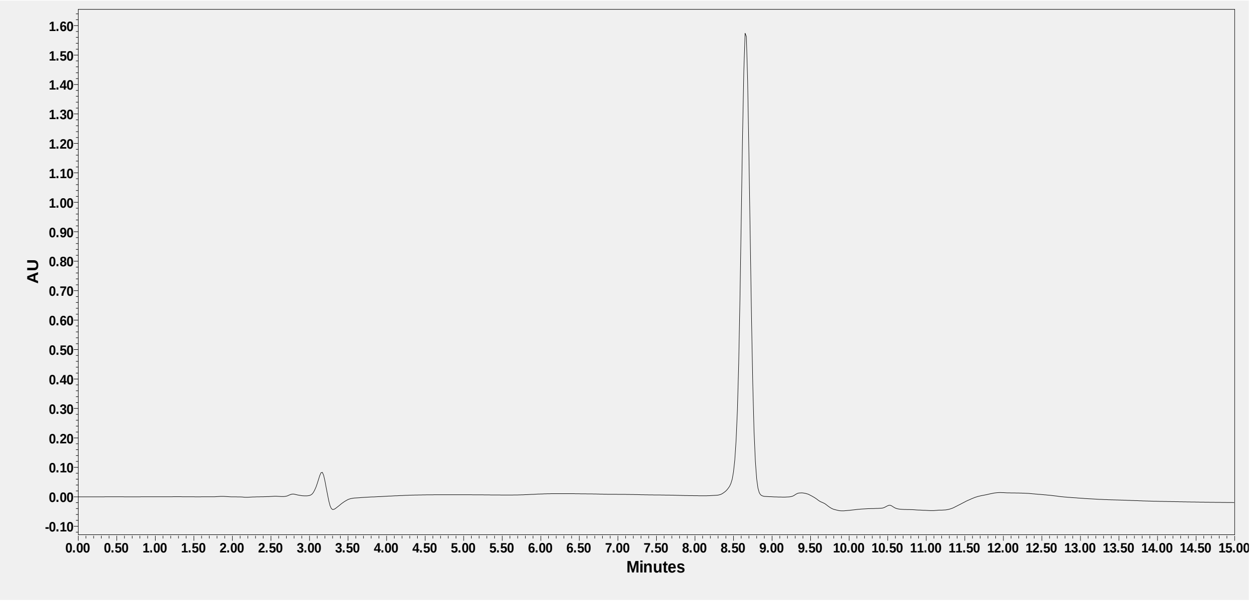

Fig. S3 The analytical HPLC chromatogram of the peptide (Ac-NH-Arg-Ala-Arg-Arg-Pro-Ser-Asn-Thr-Gln-Thr-Gln-Tyr-Ser-Ala-Cys-OH).

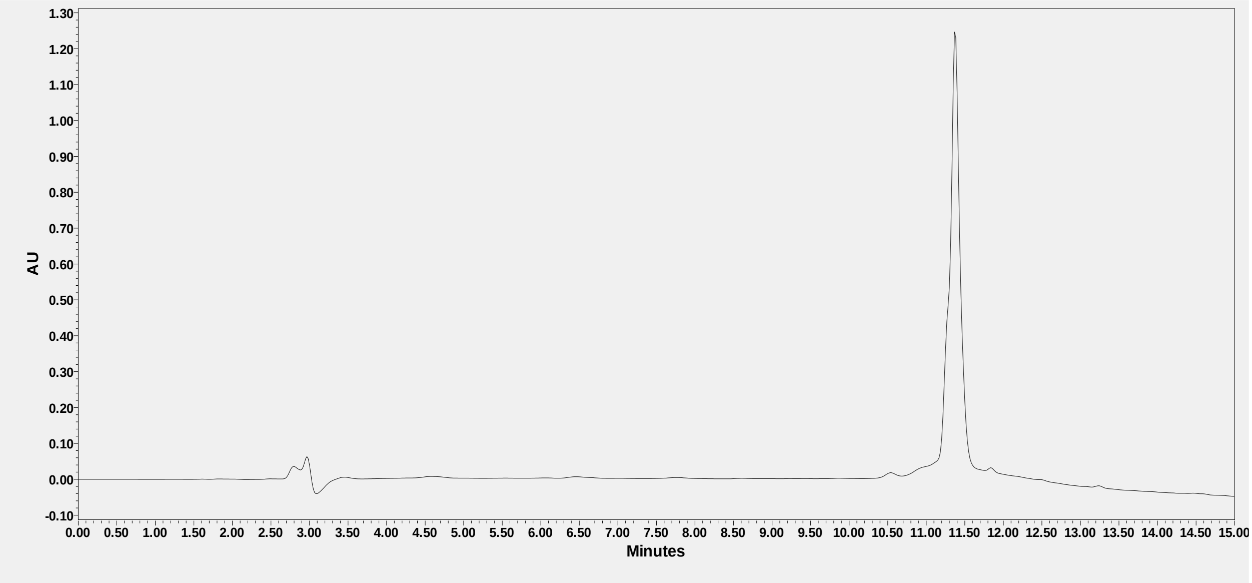

Fig. S4 The analytical HPLC chromatogram of the peptide [5(6)-FAM-NH-Arg-Ala-Arg-Arg-Pro-Ser-Asn-Thr-Gln-Thr-Gln-Tyr-Ser-Ala-Cys-OH].

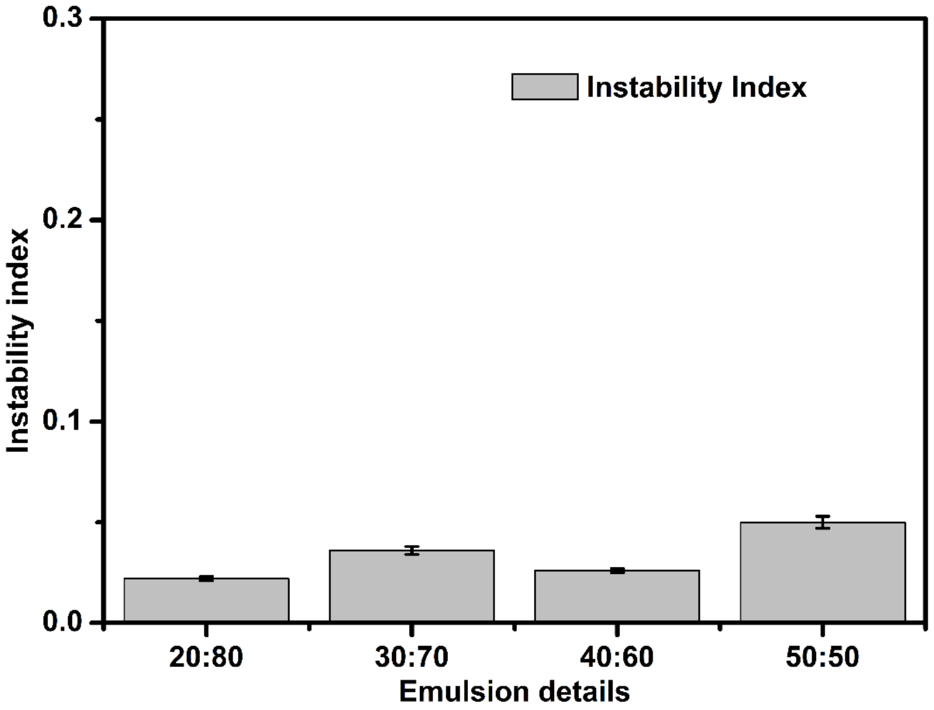

Fig. S5 Instability index of Pickering emulsions with VLP based emulsions at different oil/water ratios.

ELISA protocol for serum samples

Day 1

1. Coat three 96 well ELISA plates with 25 µL of VLP-peptide at 2.5 mg/mL (250 µg/100µL) in Carbonate/Bicarbonate Buffer.
2. Incubate the plate for 2.5 hours at 37°C.
3. Remove the coating solution and wash the plates three times with wash solution (PBS/0.05% tween), with 1-minute incubation between each wash.
4. Add 50 µL of blocking buffer (1% BSA in PBS) and incubate the plates overnight at 4°C.

Day 2

5. Remove the blocking buffer and wash the plates three times with wash solution (PBS/0.05% tween), with 1-minute incubation between each wash.
6. Add 25 µL of 1:1000, 1:10000, and 1:50000 serum samples (diluted in PBS/0.1% BSA) and blank (PBS/0.1% BSA only), in duplicates (according to plate design below) and incubate the plates overnight at 4°C.

Day 3

7. Remove the samples and wash the plates three times with wash solution (PBS/0.05% tween), with 1-minute incubation between each wash.
8. Add 25 µL of the secondary antibody and incubate for 2 hours at 37°C.
9. Remove the samples and wash the plates three times with wash solution (PBS/0.05% tween), with 1-minute incubation between each wash.
10. Add 25 µL of TMB substrate to each well and incubate for 15 min at room temperature or until the desired color is achieved.
11. Add 25 µL of Stop Solution to each well before reading the plates.
12. Read the plates at 450nm using a microplate reader.

Formulation

1. Carbonate-Bicarbonate Buffer: Empty the contents of one capsule (Sigma, Cat# C3041) in 100 mL of deionized water and dissolve. The content of one capsule yields 100 ml of 0.05 M carbonate-bicarbonate buffer, pH 9.6 at 25°C.
2. VLP-peptide: dilute 25 mg/mL VLP-peptide stock 1:10 in Carbonate/Bicarbonate Buffer.
3. Secondary Antibody (Peroxidase AffiniPure Donkey Anti-Mouse IgG (H+L) Cat 715-035-151) (1:50000 dilution in PBS/0.1% BSA)

4. Dilutions of serum: 5ul serum + 245µL buffer (1:50) and then 50ul from the 1:50 + 950µL buffer (1:1000). 100ul from the 1:1000 + 900µL buffer (1:10000). 100ul from the 1:10000 + 400µL buffer (1:50000)
5. Wash Solution– Add 100 mL of 10x PBS (pH 7.4) with 900mL of deionized water and 0.5mL Tween 20.
6. Blocking Buffer - Add 1g BSA to 100mL 1x PBS

	**Plate design (x3): one plate per serum dilution**											
	**1**	**2**	**3**	**4**	**5**	**6**	**7**	**8**	**9**	**10**	**11**	**12**
**A**	Buffer		7F		8F		9F		10F		11F	
**B**	1F		7F		8F		9F		10F		11F	
**C**	1F		7F		8F		9F		10F		12F	
**D**	3F		7F		8F		9F		11F		12F	
**E**	3F		7F		8F		10F		11F		12F	
**F**	4F		7F		9F		10F		11F		12F	
**G**	4F		8F		9F		10F		11F		12F	
**H**	7F		8F		9F		10F		11F		12F	

	**Plate design (x3): one plate per serum dilution**											
	**1**	**2**	**3**	**4**	**5**	**6**	**7**	**8**	**9**	**10**	**11**	**12**
**A**	Buffer		22		30		38		46		54	
**B**	1		23		31		39		47		55	
**C**	2		24		32		40		48		56	
**D**	13		25		33		41		49		57	
**E**	14		26		34		42		50		58	
**F**	15		27		35		43		51		59	
**G**	16		28		36		44		52		60	
**H**	21		29		37		45		53		61	
